# Epidemiology and Clinical Features of Breast Cancer in Cotonou (2014-2019): Insights from a Population-Based Registry Study

**DOI:** 10.1245/s10434-025-18976-1

**Published:** 2026-01-11

**Authors:** Freddy Houéhanou Rodrigue Gnangnon, Alexis Parenté, Togueyadji Emmanuel Allayambaye, Hélène Robin Sacca, Salmane Amidou, Benjamin Dossou-Yovo, Justie Kyria Houlda Mfoutou Mboko, Richard Biaou Boni, Cosme Toume, Véronique Blanquet, Pierre-Marie Preux, Dismand Stephan Houinato

**Affiliations:** 1 Laboratory of Epidemiology of Chronic and Neurological Diseases, LEMACEN, Cotonou, Benin; 2 Inserm U1094, IRD U270, Univ. Limoges, CHU Limoges, EpiMaCT - Epidemiology of chronic diseases in tropical zone, Institute of Epidemiology and Tropical Neurology, OmegaHealth, Limoges, France; 3 Department of Visceral Surgery, National Teaching Hospital-Hubert Koutoukou Maga, CNHU-HKM, Cotonou, Benin; 4 Department of Surgical Oncology, Faculty of Health Sciences, University of Abomey-Calavi, Cotonou, Benin; 5 National Non-Communicable Diseases Control Program, Ministry of Health, Cotonou, Republic of Benin; 6 Cotonou Cancer Registry/National Program for the Fight Against NCDs/MOH, Cotonou, Benin

**Keywords:** Breast cancer, Epidemiology, Incidence, Cancer registry, Survival, Benin

## Abstract

**Introduction:**

Breast cancer is the most frequently diagnosed cancer worldwide and a leading cause of cancer-related mortality in women. This study aimed to describe the epidemiological profile and clinical characteristics of breast cancer cases diagnosed between 2014 and 2019 among women residing in Cotonou, Benin.

**Patients and Method:**

This retrospective cohort study included 277 women with breast cancer registered in the Cotonou Population-Based Cancer Registry between 1 January 2014 and 31 December 2019. Qualitative variables were expressed as frequencies and percentages, whereas continuous variables were summarized as means with standard deviations. Overall survival was estimated using the Kaplan–Meier method. Data analyses were performed using R software (version 3.6.1) and SPSS.

**Results:**

The crude incidence of breast cancer increased from 16.8 cases per 100,000 population in 2014 to 24.7 in 2019, with a peak of 38.9 in 2018. The mean age at diagnosis was 49.6 ± 11.9 years. Most patients were diagnosed at advanced stages (III and IV), with T4 tumors being the most frequent presentation. Triple-negative breast cancer was the most prevalent molecular subtype. Overall survival was 57% at 1 year and 37% at 5 years.

**Conclusions:**

Breast cancer in Cotonou predominantly affects relatively young women and is often diagnosed at advanced stages, contributing to poor survival outcomes compared with high-income countries. Strengthening early detection and prevention strategies should be a public health priority.

Breast cancer is the leading cancer among women worldwide in both incidence and mortality, with 2,308,897 new cases and more than 665,684 deaths reported in 2022, according to the Global Cancer Observatory (GLOBOCAN 2022).^1^

The highest incidence rates are recorded in high-income countries, but 67% of deaths worldwide occur in low- and middle-income countries (LMICs), such as those in sub-Saharan Africa (SSA).^2^ In fact, although women in high-income countries have significantly higher incidence rates than those in low-income countries (54.1 versus 30.8 per 100,000), they have significantly lower mortality rates (11.3 versus 15.3 per 100,000).^1^

In sub-Saharan Africa, female breast cancer ranks first and was estimated to account for 27.3% (129,400 cases) of the 474,000 female cancer cases in 2020.^3^ It is also worth noting the absence of comprehensive data on cancer incidence and mortality in most sub-Saharan African countries due to the lack of population-based cancer registries. This represents a major obstacle to accurately estimating the cancer burden and implementing effective prevention policies, as well as to ensuring effective surveillance and monitoring, which are essential components of national cancer control plans in the region.

In Benin, the government, through the Ministry of Health and under the supervision of the National Program for NCD Control (PNLMNT), established a population-based cancer registry in Cotonou in 2014.^4^ In the country, breast cancer is known to be the leading cancer among women.^5^ In 2013, in a hospital-based survey, the overall 5-year survival rate for patients with breast cancer was 43%.^6^ In 2018, the median survival was 42 months, with a 5-year survival rate of 40% in a hospital-based setting in Benin.^7^ Data on the incidence and mortality of breast cancer from population-based studies are notably lacking in the country. This report marks an important milestone, as it presents the results from the first 6 years of registration (2014–2019). The insights gained from this comprehensive dataset not only enhance our understanding of the disease burden but also provide a foundation for evidence-based interventions and policy formulation aimed at combating breast cancer in our community.

## Patients and Materials

### Study Population

This was a retrospective, descriptive study with data collection over a 6-year period from 1 January 2014 to 31 December 2019. It focused on female breast cancer cases recorded by the Cotonou Cancer Registry (Benin). The population covered by the registry includes all individuals normally residing in the city of Cotonou, the economic capital of the Republic of Benin. The size of this population for the years 2014–2019 was estimated on the basis of the most recent population and housing census (2013).^4^ The average population of the country between 2014 and 2019 was 11,884,127, comprising 6,037,577 women and 5,846,550 men.^8^

### Collection and Management of Data

The Cotonou Cancer Registry follows the Standard Operating Procedures Manual of the African Cancer Registry Network (AFCRN). Cancer cases are registered regardless of the diagnostic basis (pathology, imaging, tumor markers, or clinical examination). Data collection is active in approximately 28 sources where patients with cancer residing in Cotonou (the economic capital city of the Republic of Benin) may be diagnosed and/or treated: 9 public health facilities, 14 private clinics, and 5 pathology laboratories.^4^ During the study period, the average population of the Littoral Department was estimated at 719,712 inhabitants (378,745 women and 340,967 men), representing 6.8% of Benin’s total population.^4,9^

The Centre National Hospitalier Universitaire Hubert Koutoukou Maga (CNHU-HKM) and the Centre Hospitalier Universitaire de la Mère et de l’Enfant Lagune (CHU-MEL), two tertiary-level hospitals and national referral centers for breast cancer management, are the registry’s primary sources of information for patients with breast cancer. Appropriate data collection methods have been established for each source. Information on cancer cases (patient personal data, tumor details and treatment, source of information, and patient outcome, alive or deceased) is extracted from source records using data collection forms. Data are coded [using the International Classification of Diseases for Oncology, third edition (ICD-O-3), for tumor site and morphology] before being entered into a database managed with CanReg5 software, developed by the International Agency for Research on Cancer (IARC).^4^

### Data Quality and Completeness

Data collection, quality control, and consistency are ensured using DepEdit and IARCTools, which are integrated into the CanReg5 system.

### Vital Status

Vital status was obtained through active methods. Clinical records were reviewed, and the patient’s vital status at the closing date was recorded. Cases whose vital status could not be confirmed through this process were contacted by phone when a mobile number was available in the registry record. When no further information could be obtained, home visits were conducted by registry staff. Patients whose vital status (alive or deceased) could not be determined by the study’s closing date were censored.

### Ethics Approval and Consent to Participate

The study was approved by the Health Sciences Research and Ethics Committee in Cotonou, Benin (no. 005 2021/UAC/CERSS/P/SG/R/SA). Informed consent was obtained from all participants and/or their legal guardians. The study was conducted in accordance with the Declaration of Helsinki. Strict security measures were implemented to ensure confidentiality. Completed forms were securely stored in a locked cabinet at the Laboratory of Epidemiology of Chronic and Neurological Diseases (LEMACEN) at the Faculty of Health Sciences, with access restricted to authorized personnel only.

### Data Analysis

Data analysis was performed using R and SPSS software. Qualitative variables were expressed as numbers and percentages, while quantitative variables were described using means and standard deviations. Population standardization was performed according to the World Health Organization’s standard population.^10^ Survival was estimated and described using the Kaplan–Meier method.

## Results

### Incidence and Burden of Breast Cancer

**Fig. 1:**
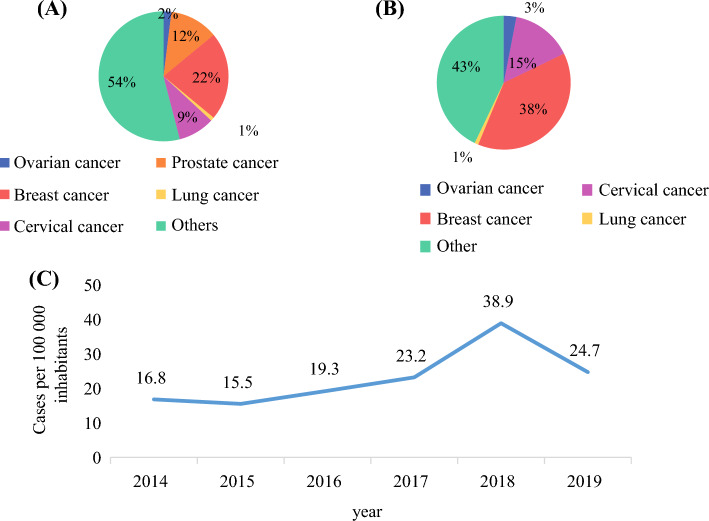
Data from the Cotonou Population-Based Cancer Registry, 2014–2019: **A** most frequent cancers in both sexes, **B** most frequent cancers in female individuals, **C** crude incidence rates of breast cancer

Between 2014 and 2019, the Cotonou Cancer Registry recorded 541 cases of breast cancer, including 13 cases in men. Breast cancer was the most common cancer diagnosed in Cotonou in both sexes (22%) and the most common cancer in women (38%) (Fig. 1A, B). The crude incidence rate of breast cancer ranged from 16.8 cases per 100,000 population in 2014 to 24.7 cases per 100,000 in 2019, with a peak of 38.9 cases per 100,000 in 2018 (Fig. 1C). The age-standardized incidence rate (ASR) per 100,000 population ranged from 22.8 in 2014 to 45.9 in 2019, with a peak of 59.3 in 2018 (Table 1).

**Table 1:** Crude and age-standardized incidence rates of female breast cancer (per 100,000 population) in Cotonou, 2014–2019

Ages/years	< 20	< 35	< 45	< 55	< 65	< 75	80+	Crude rate	ASR
2014	5.4	31.7	118.1	145.3	128.3	105.3	0.0	16.8	22.8
2015	2.7	42.1	109.1	132.8	79.8	188.6	130.5	15.5	22.0
2016	0.0	65.1	127.6	132.8	145.4	182.5	0.0	19.3	26.0
2017	0.0	77.9	157.0	178.6	155.5	110.7	0.0	23.2	30.3
2018	0.0	73.3	280.0	291.2	452.6	230.9	287.4	38.9	59.3
2019	0.0	51.6	144.0	268.5	449.5	190.8	66.7	24.7	45.9

### Characteristics of Patients with Breast Cancer

We were able to trace the medical records of 277 out of the 528 female patients listed in the Cotonou Cancer Registry. Age at diagnosis ranged from 20 to 91 years, with a mean of 49.6 ± 11.9 years (Table 2). Approximately 54.2% of patients were under 50 years old at the time of diagnosis. Most patients with breast cancer were either shopkeepers (24.9%) or homemakers (19.5%). With respect to education, the highest proportion had no formal education (28.8%), followed by patients with secondary education (25.6%). Most patients lived in urban areas (88.4%) (Table 3).

**Table 2:** Sociodemographic characteristics of women diagnosed with breast cancer in Cotonou, 2014–2019

Characteristics (*n* = 277)	Frequency	%
*Age (years)*		
*Mean ± SD: 49.64 ± 11.89*		
< 30	6	2.2
30–40	40	14.4
40–50	104	37.5
50–60	68	24.5
≥ 60	59	21.3
*Occupation*		
Student	1	0.3
Unemployed	1	0.3
Housemaker	54	19.5
Manual worker	32	11.5
Shopkeeper	69	24.9
Middle management	41	14.8
Senior manager	15	5.4
Not specified	64	23.0
*Education level*		
None	23	8.3
Primary	62	22.4
Secondary	71	25.6
Tertiary	41	14.8
Not specified	80	28.8
*Residence area*		
Urban	245	88.4
Rural	14	5.1
Not specified	18	6.5

**Table 3:** Clinical and tumor characteristics of women diagnosed with breast cancer in Cotonou, 2014–2019

Laterality	Frequency	(%)
Right	135	49.0
Left	108	37.2
Bilateral	9	3.2
Not specified	25	10.6
*TNM Classification*		
T0	2	0.7
T1	14	5.0
T2	48	17.3
T3	45	16.2
T4	115	41.5
Tx	28	10.1
Not specified	25	9.0
N0	50	18.0
N1	93	33.5
N2	78	28.1
N3	16	5.7
Nx	17	6.1
Not specified	23	8.3
M0	138	49.8
M1	66	23.8
Mx	48	17.3
Not specified	25	9.1

SBR	Frequency	(%)
SBR1	13	4.7
SBR2	86	31.0
SBR3	50	18.1
Not specified	128	46.2
*UICC*		
Stage 0	3	1.1
Stage I	20	7.2
Stage II	29	10.5
Stage III	91	32.8
Stage IV	65	23.5
Not specified	69	24.9
*Molecular subtype*		
Her2	17	6.1
Unclassifiable	3	1.1
Luminal A	41	14.8
Luminal B not Her	23	8.3
Luminal B non Her +	11	3.9
*Triple negative*	49	17.7
Not specified	133	48.0
*Radiotherapy performed*		
Yes	32	115
No	145	52.3
Not specified	100	36.1
*Chemotherapy performed*		
Yes	178	64.3
No	37	13.4
Not specified	62	22.4
*Surgery*		
Yes	144	51.9
No	61	22.0
Not specified	72	25.9
*Hormone therapy performed*		
Yes	4	1.4
No	143	51.6
Not specified	130	46.9

### Clinical and Therapeutic Characteristics

**Fig. 2:**
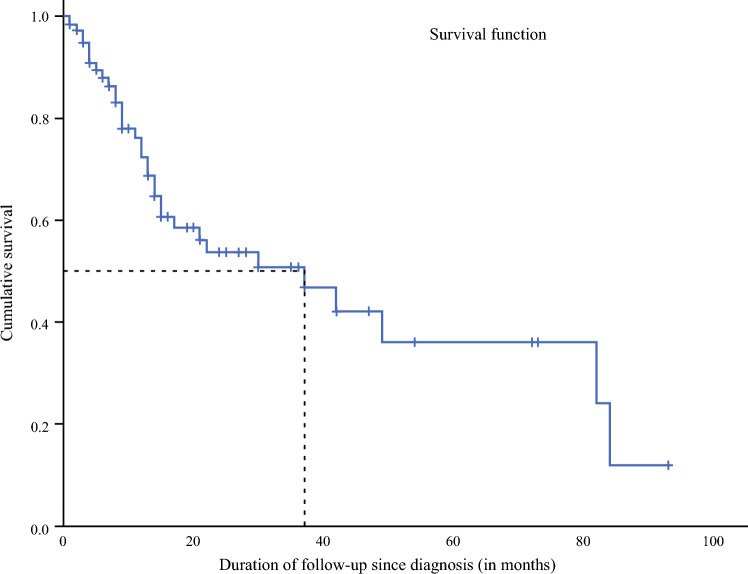
Overall survival of women diagnosed with breast cancer in Cotonou between 2014 and 2019

Tumors were predominantly diagnosed at advanced stages; among the 75.1% of cases with available staging, 32.8% were classified as stage III and 23.5% as stage IV (Fig. 2). The Scarff–Bloom–Richardson (SBR) grading system was used for evaluation in 53.8% of cases, with 4.7% classified as grade I, 31.0% as grade II, and 18.1% as grade III. Immunohistochemistry results were available for 144 patients, of whom 49 had triple-negative breast cancers and 41 had luminal A breast cancers (Table 3). Targeted therapy was the least common type of treatment, followed by radiotherapy (Table 3).

### Overall Survival

The 1-year overall survival rate was 57%, decreasing slightly to 54% at 2 years, then declining to 44% at 3 years, and further to 37% at 5 years. The median survival time was 37 months.

## Discussion

In low- and middle-income countries (LMICs) such as Benin, where healthcare facilities are often limited or scarce, the quality of cancer registry data may be compromised.^11^ However, data from the Cotonou Cancer Registry for the period 2015–2017 were included in the twelfth volume of Cancer Incidence in Five Continents.^12^ Cancer Incidence in Five Continents (CI5) is a joint initiative of the International Agency for Research on Cancer (IARC) and International Association of Cancer Registries (IACR), published every 5 years, which compiles high-quality data from population-based cancer registries worldwide.^13^

In our study, the crude incidence of breast cancer ranged from 16.8 per 100,000 in 2014 to 25.0 in 2019. This is lower than the 34.25 per 100,000 reported in Tunisia^14^ in 2022 and markedly below the rates in Brazil,^15^ where white and Black women had median incidences of 101.3 and 59.7 per 100,000, respectively. Higher breast cancer incidence in transitioned settings, such as Brazil, is associated with reproductive and lifestyle factors including delayed childbirth, low parity, reduced breastfeeding, use of hormonal therapies, alcohol consumption, obesity, and physical inactivity.^1,16^

The mean age at diagnosis in our cohort was 49.6 years, similar to 45.8 years in Ethiopia^17^ and slightly lower than 52.83 years in Madagascar.^18^ In France (2020), Dabakuyo-Yonli et al. reported a mean age at breast cancer diagnosis of 63 years.^19^ The relatively young age at breast cancer diagnosis observed in developing countries, such as those in sub-Saharan Africa, may be explained by three main hypotheses: (1) biological differences in tumors, (2) a cohort effect related to the high proportion of young individuals in these populations, and (3) underreporting or incomplete cancer registration among older women.^20^

In this study, most patients with breast cancer belonged to disadvantaged socioeconomic groups. Traditionally, breast cancer has been associated with higher socioeconomic position (SEP) in high-income countries, partly due to reproductive patterns, hormonal exposures, and access to screening services. However, evidence from sub-Saharan Africa challenges this paradigm. Similar to our findings, the large multicountry African Breast Cancer–Disparities in Outcomes (ABC-DO) cohort reported that the majority of women were of low socioeconomic position, with most of them engaged in unskilled occupations.^21^ These patterns may in part be explained by the relatively high proportion of women living in disadvantaged socioeconomic conditions in sub-Saharan Africa.

In this study, the average consultation delay was 8.1 months, similar to the 8.1 months observed in Dakar, Senegal, in 2008.^22^ Delayed consultation after breast symptoms in SSA is mainly due to ignorance, belief in spiritual healing, fear of mastectomy, and reliance on herbal treatment, highlighting the need to target high-risk women in awareness campaigns.^23^

In our study, 57.7% (160 out of 277 cases) of breast cancers were diagnosed at stages T3 and T4. Several studies in sub-Saharan Africa have reported high proportions of advanced-stage breast cancer at diagnosis, with rates reaching 76–89% for stages T3 and T4 in Cameroon^24^ and Ivory Coast.^25^ In contrast, countries with higher socioeconomic levels, such as Morocco^26^ and Croatia,^27^ report much lower rates of advanced-stage tumors.

Lymph node involvement was frequent in our study (67.3%), with comparable rates in Cameroon (73.6%), lower in Ivory Coast (49.5%), and much lower in Morocco (38.9%). In our study, 24% of tumors were metastatic at diagnosis, versus 18.4% in a multicountry sub-Saharan African study,^28^ and about 5–6% in France, Italy,^29^ and the USA.^30^

Late-stage diagnosis of breast cancer in sub-Saharan Africa results from multiple factors, including the absence of national screening programs due to limited infrastructure, financial constraints, and workforce shortages.^31,32^ Low health literacy, shaped by limited educational opportunities, cultural stigma, misconceptions, and misinformation, further impedes early detection.^23^ Geographic and economic barriers, such as long travel distances, high out-of-pocket costs, and weak health systems, also contribute, as many patients initially seek care from traditional healers or use herbal remedies, thereby increasing the likelihood of advanced-stage presentation.^32^

In this study, the treatment patterns observed reflect the complex interplay between late-stage presentation and the limited availability of cancer care services in the country.^33^ While the relatively high proportion of women who received chemotherapy can be explained by the high frequency of advanced-stage disease and the predominance of triple-negative and HER2-overexpressing tumors—subtypes for which chemotherapy is the first-line treatment—the proportion of patients who underwent surgery (51.9%) appears relatively low. This finding highlights the limited access to surgical care in our setting, despite surgery being the most widely available treatment modality for breast cancer in sub-Saharan Africa. Beyond financial constraints, fear of mastectomy is another significant factor limiting access to surgery.^33,34^ In our clinical practice, it is not uncommon to observe that some patients—especially those who show a good response to neoadjuvant chemotherapy—subsequently refuse surgery and are lost to follow-up, returning only upon disease progression. These observations underscore the substantial influence of economic and sociocultural determinants on therapeutic pathways. In fact, in low- and middle-income countries such as Benin, many patients reach the surgeon only after the disease has progressed to a locally advanced or metastatic stage. This situation largely explains the low rates of breast-conserving surgery and the high rates of axillary lymph node dissection reported in breast cancer series from Benin, and more broadly, across sub-Saharan Africa.^35^

Radiotherapy was administered to only 11.5% of patients, a proportion that is very low given the high burden of locally advanced breast cancers (stage III), triple-negative tumors, and HER2-positive disease. This reflects the limited radiotherapy capacity in the country, as Benin did not have a fully functional radiotherapy unit until 2025. Insufficient access to radiotherapy remains one of the major barriers to the continuum of care for breast cancer in sub-Saharan Africa and a key obstacle to surgical de-escalation, as radiotherapy is essential for breast-conserving treatment.

Immunotherapy is not currently available in Benin, and access to targeted therapies such as trastuzumab remains extremely limited due to their prohibitive costs and lack of reimbursement.^36^ In sub-Saharan Africa, one of the major barriers to cancer treatment is still the prohibitive cost of care for patients and their families.^37^ These financial and structural barriers further exacerbate inequities in breast cancer care, leaving many patients without access to potentially life-saving treatments.^36^

In this study, triple-negative breast cancer was the most common subtype, followed by luminal A, consistent with findings from several sub-Saharan African countries.^38,39^ By contrast, studies from North Africa,^40^ Saudi Arabia,^41^ and the USA^42^ reported luminal subtypes as predominant, with triple-negative cases being less frequent. The high frequency of triple-negative breast cancer (TNBC) in sub-Saharan Africa may be explained by three main factors. First is genetic susceptibility, as studies have shown gene subsets and immune profiles specific to women of African origin.^43^ Second is the younger age structure of African populations, since younger age at diagnosis (< 40 years) has been linked to TNBC.^44^ Third are possible false negatives in immunohistochemistry, with up to 20% of results worldwide estimated to be inaccurate due to pre-analytical variables.^45^

The overall survival rate in our study was 57% at 1 year, 54% at 2 years, 44% at 3 years, and 37% at 5 years. Breast cancer survival in sub-Saharan Africa remains poor, with 5-year rates ranging from 11% in Ethiopia^46^ to 48% in Benin,^36^ compared with about 88% in France.^47^ Joko-Fru et al., in a retrospective study conducted in 2019 in 12 African countries, found an overall survival rate of 84.1% at 1 year and 61.4% at 3 years.^28^ These disparities reflect differences in stage at diagnosis, tumor biology, access to treatment, and early detection policies.

Thus, beyond individual clinical factors, it is important to situate our findings within the broader context of national cancer control policies in Benin and the wider sub-Saharan African (SSA) region.^31^ In Benin, population-based breast cancer screening programs are not yet implemented; early detection relies largely on opportunistic clinical breast examinations, and diagnostic delays remain frequent because of limited imaging and pathology capacity. Treatment services are also concentrated in urban tertiary hospitals,^48^ and high out-of-pocket costs for chemotherapy, targeted therapy, and supportive care substantially limit access to timely and optimal treatment.^31^

As shown in our study from the Cotonou Population-Based Cancer Registry, most breast cancers were diagnosed at advanced stages (III and IV), with 57.7% of patients presenting with T3–T4 tumors and 24% already metastatic at diagnosis. These findings illustrate the critical consequences of lacking structured screening and early detection policies, which contribute to delayed presentation and poor survival. Strengthening national cancer control plans with effective, resource-appropriate screening and early detection strategies, aligned with the World Health Organization (WHO) Global Breast Cancer Initiative (GBCI),^49^ is therefore an urgent public health priority in Benin and across the sub-Saharan African region.^21^

## Conclusions

Enhancing breast cancer survival in Benin is a pressing priority. It appears that the incidence of breast cancer among women in Cotonou, the capital city, is on the rise (from 16.8 cases per 100,000 in 2014 to 38.9 cases per 100,000 in 2019) and affects a much younger population. Triple-negative breast cancer was the most prevalent type in the study population. The overall survival of women with breast cancer in Cotonou remains poorer compared with developed countries despite advances in oncology. Although the incidence of breast cancer in the region is lower than in developed countries, the proportion of late-stage cases is higher, largely due to the absence of effective screening policies, delayed access to care, insufficient technical resources, and limited healthcare access. To improve the survival of women with breast cancer in Benin, it is both important and urgent to implement effective screening and early diagnosis policies, conduct extensive awareness campaigns, facilitate access to care, and adequately equip healthcare facilities.

## Data Availability

The datasets used and/or analyzed during the current study are available from the corresponding author upon reasonable request.
